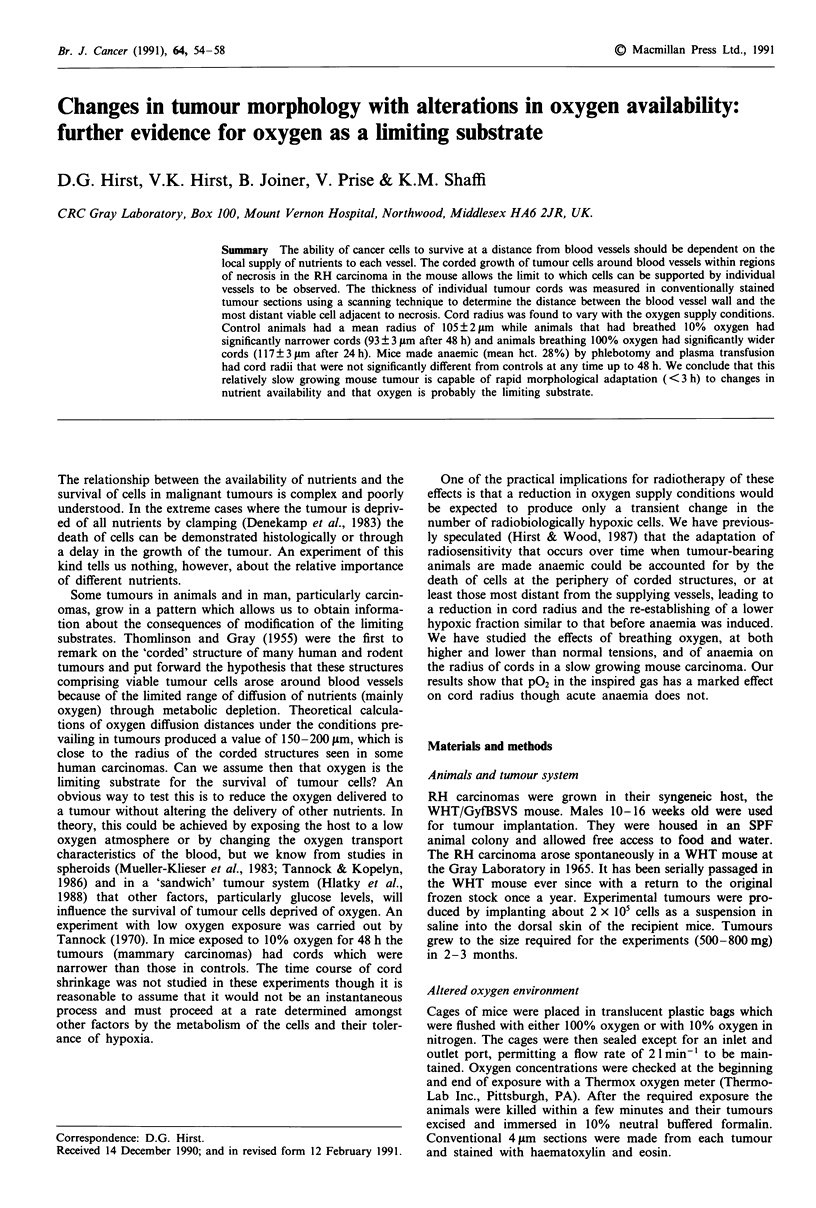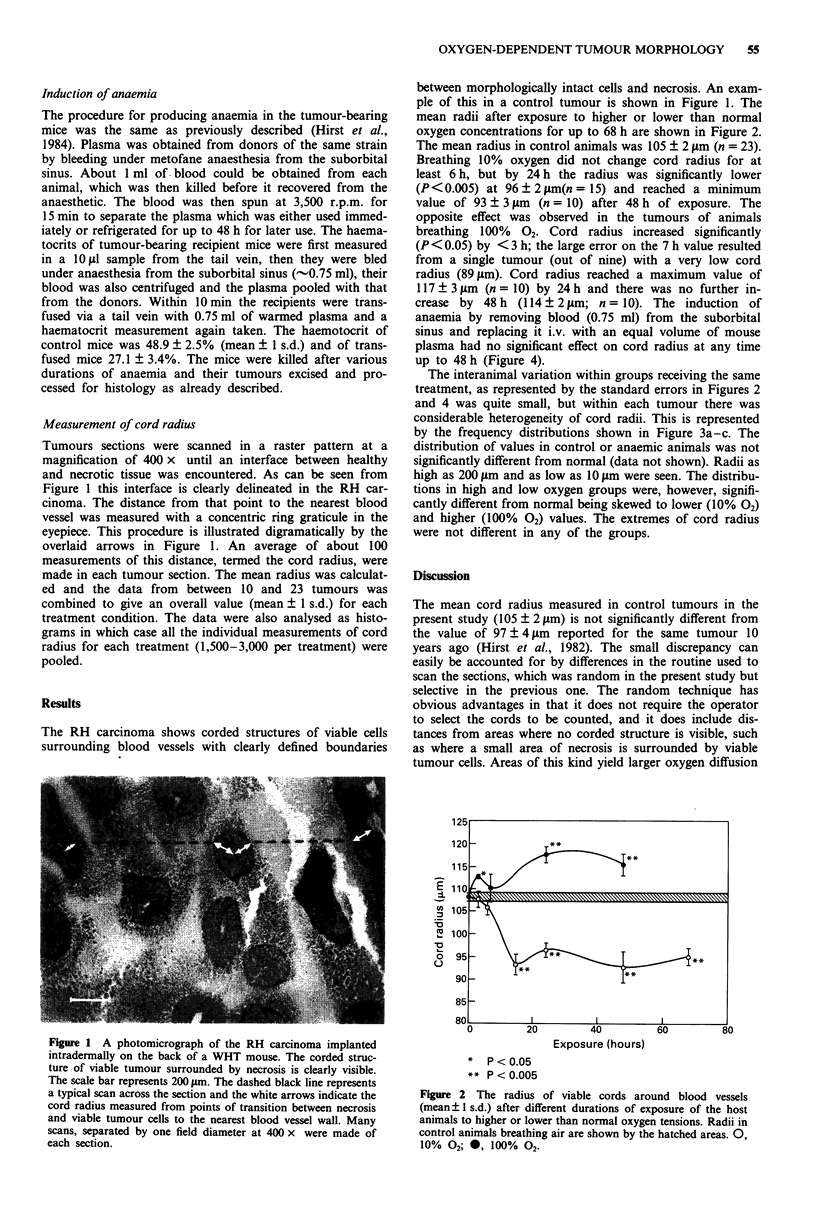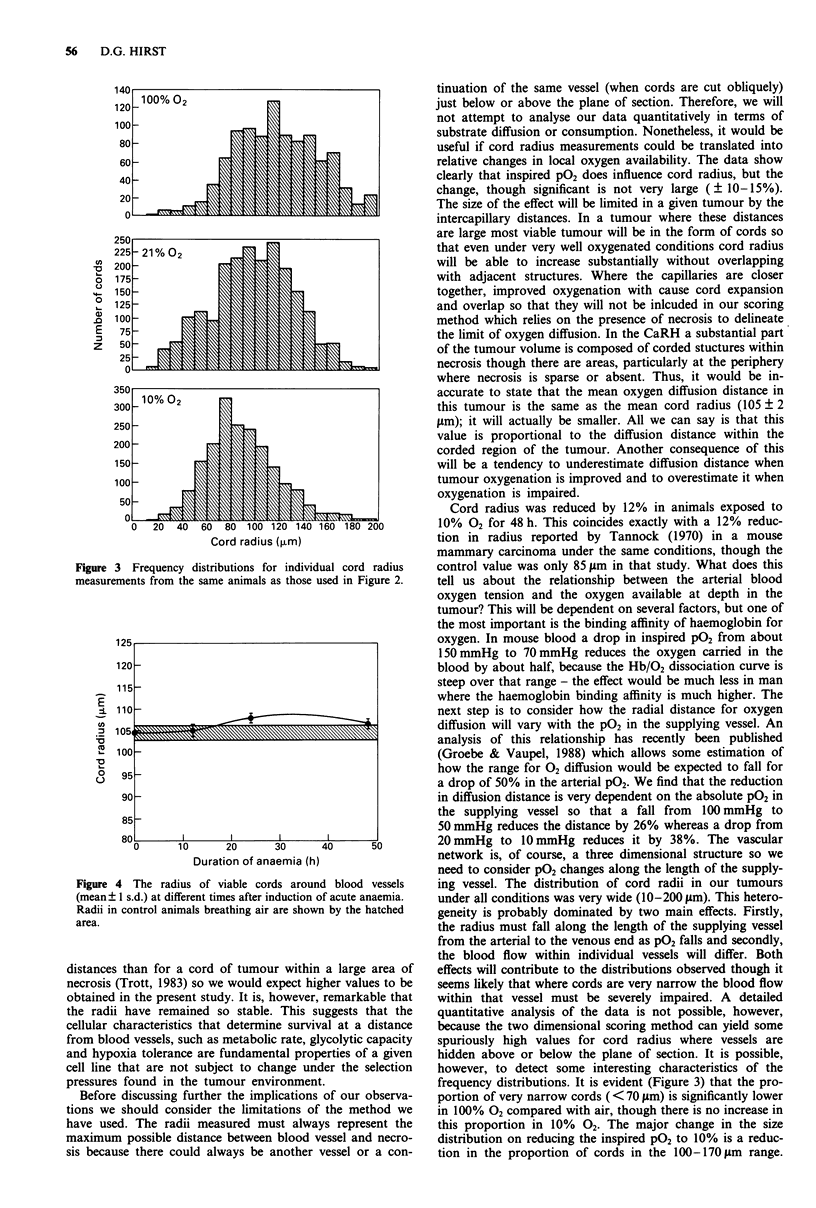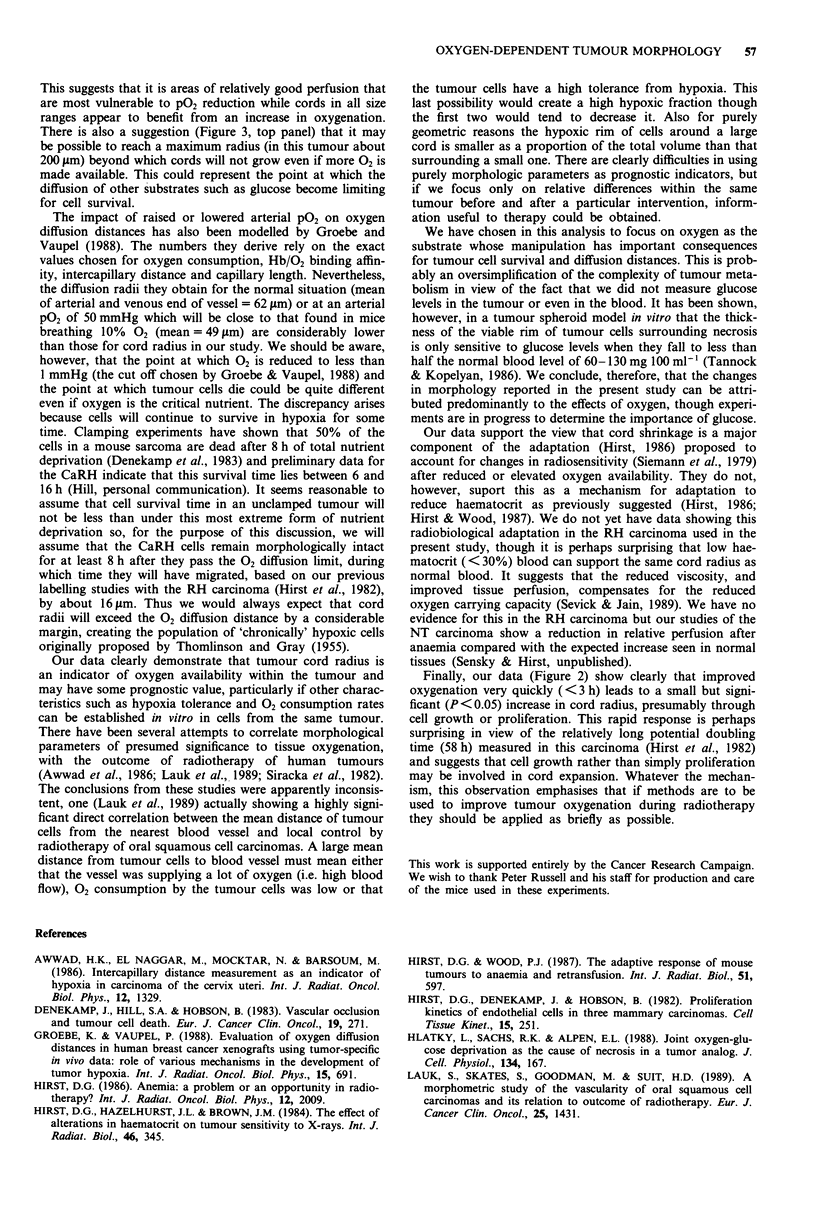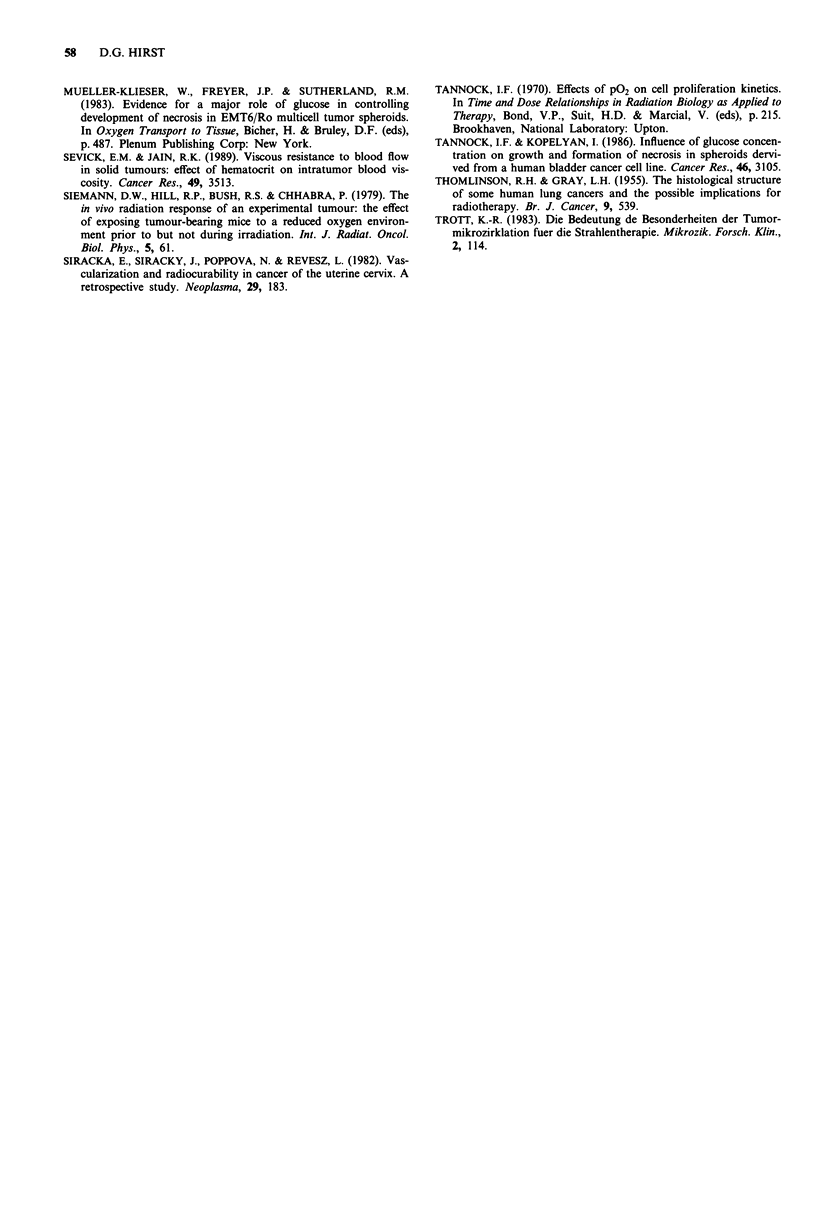# Changes in tumour morphology with alterations in oxygen availability: further evidence for oxygen as a limiting substrate.

**DOI:** 10.1038/bjc.1991.238

**Published:** 1991-07

**Authors:** D. G. Hirst, V. K. Hirst, B. Joiner, V. Prise, K. M. Shaffi

**Affiliations:** CRC Gray Laboratory, Mount Vernon Hospital, Northwood, Middlesex, UK.

## Abstract

**Images:**


*Br. J. Cancer* (1991), **64**, 54–58 © Macmillan Press Ltd., 1991

### Changes in tumour morphology with alterations in oxygen availability: further evidence for oxygen as a limiting substrate

D.G. Hirst, V.K. Hirst, B. Joiner, V. Prise & K.M. Shaffi

*CRC Gray Laboratory, Box 100, Mount Vernon Hospital, Northwood, Middlesex HA6 2JR, UK.*

**Summary** The ability of cancer cells to survive at a distance from blood vessels should be dependent on the local supply of nutrients to each vessel. The corded growth of tumour cells around blood vessels within regions of necrosis in the RH carcinoma in the mouse allows the limit to which cells can be supported by individual vessels to be observed. The thickness of individual tumour cords was measured in conventionally stained tumour sections using a scanning technique to determine the distance between the blood vessel wall and the most distant viable cell adjacent to necrosis. Cord radius was found to vary with the oxygen supply conditions. Control animals had a mean radius of $105 \pm 2\,\mu m$ while animals that had breathed 10% oxygen had significantly narrower cords ($93 \pm 3\,\mu m$ after 48 h) and animals breathing 100% oxygen had significantly wider cords ($117 \pm 3\,\mu m$ after 24 h). Mice made anaemic (mean hct. 28%) by phlebotomy and plasma transfusion had cord radii that were not significantly different from controls at any time up to 48 h. We conclude that this relatively slow growing mouse tumour is capable of rapid morphological adaptation ($<3$ h) to changes in nutrient availability and that oxygen is probably the limiting substrate.

The relationship between the availability of nutrients and the survival of cells in malignant tumours is complex and poorly understood. In the extreme cases where the tumour is deprived of all nutrients by clamping (Denekamp *et al.*, 1983) the death of cells can be demonstrated histologically or through a delay in the growth of the tumour. An experiment of this kind tells us nothing, however, about the relative importance of different nutrients.

Some tumours in animals and in man, particularly carcinomas, grow in a pattern which allows us to obtain information about the consequences of modification of the limiting substrates. Thomlinson and Gray (1955) were the first to remark on the 'corded' structure of many human and rodent tumours and put forward the hypothesis that these structures comprising viable tumour cells arose around blood vessels because of the limited range of diffusion of nutrients (mainly oxygen) through metabolic depletion. Theoretical calculations of oxygen diffusion distances under the conditions prevailing in tumours produced a value of 150–200 µm, which is close to the radius of the corded structures seen in some human carcinomas. Can we assume then that oxygen is the limiting substrate for the survival of tumour cells? An obvious way to test this is to reduce the oxygen delivered to a tumour without altering the delivery of other nutrients. In theory, this could be achieved by exposing the host to a low oxygen atmosphere or by changing the oxygen transport characteristics of the blood, but we know from studies in spheroids (Mueller-Klieser *et al.*, 1983; Tannock & Kopelyn, 1986) and in a 'sandwich' tumour system (Hlatky *et al.*, 1988) that other factors, particularly glucose levels, will influence the survival of tumour cells deprived of oxygen. An experiment with low oxygen exposure was carried out by Tannock (1970). In mice exposed to 10% oxygen for 48 h the tumours (mammary carcinomas) had cords which were narrower than those in controls. The time course of cord shrinkage was not studied in these experiments though it is reasonable to assume that it would not be an instantaneous process and must proceed at a rate determined amongst other factors by the metabolism of the cells and their tolerance of hypoxia.

Correspondence: D.G. Hirst.
Received 14 December 1990; and in revised form 12 February 1991.

One of the practical implications for radiotherapy of these effects is that a reduction in oxygen supply conditions would be expected to produce only a transient change in the number of radiobiologically hypoxic cells. We have previously speculated (Hirst & Wood, 1987) that the adaptation of radiosensitivity that occurs over time when tumour-bearing animals are made anaemic could be accounted for by the death of cells at the periphery of corded structures, or at least those most distant from the supplying vessels, leading to a reduction in cord radius and the re-establishing of a lower hypoxic fraction similar to that before anaemia was induced. We have studied the effects of breathing oxygen, at both higher and lower than normal tensions, and of anaemia on the radius of cords in a slow growing mouse carcinoma. Our results show that $pO_2$ in the inspired gas has a marked effect on cord radius though acute anaemia does not.

### Materials and methods

### *Animals and tumour system*

RH carcinomas were grown in their syngeneic host, the WHT/GyfBSVS mouse. Males 10–16 weeks old were used for tumour implantation. They were housed in an SPF animal colony and allowed free access to food and water. The RH carcinoma arose spontaneously in a WHT mouse at the Gray Laboratory in 1965. It has been serially passaged in the WHT mouse ever since with a return to the original frozen stock once a year. Experimental tumours were produced by implanting about $2 \times 10^5$ cells as a suspension in saline into the dorsal skin of the recipient mice. Tumours grew to the size required for the experiments (500–800 mg) in 2–3 months.

### *Altered oxygen environment*

Cages of mice were placed in translucent plastic bags which were flushed with either 100% oxygen or with 10% oxygen in nitrogen. The cages were then sealed except for an inlet and outlet port, permitting a flow rate of $2\,l\,min^{-1}$ to be maintained. Oxygen concentrations were checked at the beginning and end of exposure with a Thermox oxygen meter (Thermo-Lab Inc., Pittsburgh, PA). After the required exposure the animals were killed within a few minutes and their tumours excised and immersed in 10% neutral buffered formalin. Conventional 4 µm sections were made from each tumour and stained with haematoxylin and eosin.

*Induction of anaemia*

The procedure for producing anaemia in the tumour-bearing mice was the same as previously described (Hirst *et al.*, 1984). Plasma was obtained from donors of the same strain by bleeding under metofane anaesthesia from the suborbital sinus. About 1 ml of blood could be obtained from each animal, which was then killed before it recovered from the anaesthetic. The blood was then spun at 3,500 r.p.m. for 15 min to separate the plasma which was either used immediately or refrigerated for up to 48 h for later use. The haematocrits of tumour-bearing recipient mice were first measured in a 10 μl sample from the tail vein, then they were bled under anaesthesia from the suborbital sinus (~0.75 ml), their blood was also centrifuged and the plasma pooled with that from the donors. Within 10 min the recipients were transfused via a tail vein with 0.75 ml of warmed plasma and a haematocrit measurement again taken. The haemotocrit of control mice was 48.9 ± 2.5% (mean ± 1 s.d.) and of transfused mice 27.1 ± 3.4%. The mice were killed after various durations of anaemia and their tumours excised and processed for histology as already described.

*Measurement of cord radius*

Tumours sections were scanned in a raster pattern at a magnification of 400 × until an interface between healthy and necrotic tissue was encountered. As can be seen from Figure 1 this interface is clearly delineated in the RH carcinoma. The distance from that point to the nearest blood vessel was measured with a concentric ring graticule in the eyepiece. This procedure is illustrated digramatically by the overlaid arrows in Figure 1. An average of about 100 measurements of this distance, termed the cord radius, were made in each tumour section. The mean radius was calculated and the data from between 10 and 23 tumours was combined to give an overall value (mean ± 1 s.d.) for each treatment condition. The data were also analysed as histograms in which case all the individual measurements of cord radius for each treatment (1,500–3,000 per treatment) were pooled.

### Results

The RH carcinoma shows corded structures of viable cells surrounding blood vessels with clearly defined boundaries between morphologically intact cells and necrosis. An example of this in a control tumour is shown in Figure 1. The mean radii after exposure to higher or lower than normal oxygen concentrations for up to 68 h are shown in Figure 2. The mean radius in control animals was 105 ± 2 μm ($n = 23$). Breathing 10% oxygen did not change cord radius for at least 6 h, but by 24 h the radius was significantly lower ($P < 0.005$) at 96 ± 2 μm($n = 15$) and reached a minimum value of 93 ± 3 μm ($n = 10$) after 48 h of exposure. The opposite effect was observed in the tumours of animals breathing 100% $O_2$. Cord radius increased significantly ($P < 0.05$) by < 3 h; the large error on the 7 h value resulted from a single tumour (out of nine) with a very low cord radius (89 μm). Cord radius reached a maximum value of 117 ± 3 μm ($n = 10$) by 24 h and there was no further increase by 48 h (114 ± 2 μm; $n = 10$). The induction of anaemia by removing blood (0.75 ml) from the suborbital sinus and replacing it i.v. with an equal volume of mouse plasma had no significant effect on cord radius at any time up to 48 h (Figure 4).

The interanimal variation within groups receiving the same treatment, as represented by the standard errors in Figures 2 and 4 was quite small, but within each tumour there was considerable heterogeneity of cord radii. This is represented by the frequency distributions shown in Figure 3a–c. The distribution of values in control or anaemic animals was not significantly different from normal (data not shown). Radii as high as 200 μm and as low as 10 μm were seen. The distributions in high and low oxygen groups were, however, significantly different from normal being skewed to lower (10% $O_2$) and higher (100% $O_2$) values. The extremes of cord radius were not different in any of the groups.

### Discussion

The mean cord radius measured in control tumours in the present study (105 ± 2 μm) is not significantly different from the value of 97 ± 4 μm reported for the same tumour 10 years ago (Hirst *et al.*, 1982). The small discrepancy can easily be accounted for by differences in the routine used to scan the sections, which was random in the present study but selective in the previous one. The random technique has obvious advantages in that it does not require the operator to select the cords to be counted, and it does include distances from areas where no corded structure is visible, such as where a small area of necrosis is surrounded by viable tumour cells. Areas of this kind yield larger oxygen diffusion

**Figure 1** A photomicrograph of the RH carcinoma implanted intradermally on the back of a WHT mouse. The corded structure of viable tumour surrounded by necrosis is clearly visible. The scale bar represents 200 μm. The dashed black line represents a typical scan across the section and the white arrows indicate the cord radius measured from points of transition between necrosis and viable tumour cells to the nearest blood vessel wall. Many scans, separated by one field diameter at 400 × were made of each section.

**Figure 2** The radius of viable cords around blood vessels (mean ± 1 s.d.) after different durations of exposure of the host animals to higher or lower than normal oxygen tensions. Radii in control animals breathing air are shown by the hatched areas. ○, 10% $O_2$; ●, 100% $O_2$.

**Figure 3** Frequency distributions for individual cord radius measurements from the same animals as those used in Figure 2.

**Figure 4** The radius of viable cords around blood vessels (mean ± 1 s.d.) at different times after induction of acute anaemia. Radii in control animals breathing air are shown by the hatched area.

distances than for a cord of tumour within a large area of necrosis (Trott, 1983) so we would expect higher values to be obtained in the present study. It is, however, remarkable that the radii have remained so stable. This suggests that the cellular characteristics that determine survival at a distance from blood vessels, such as metabolic rate, glycolytic capacity and hypoxia tolerance are fundamental properties of a given cell line that are not subject to change under the selection pressures found in the tumour environment.

Before discussing further the implications of our observations we should consider the limitations of the method we have used. The radii measured must always represent the maximum possible distance between blood vessel and necrosis because there could always be another vessel or a continuation of the same vessel (when cords are cut obliquely) just below or above the plane of section. Therefore, we will not attempt to analyse our data quantitatively in terms of substrate diffusion or consumption. Nonetheless, it would be useful if cord radius measurements could be translated into relative changes in local oxygen availability. The data show clearly that inspired $pO_2$ does influence cord radius, but the change, though significant is not very large ($\pm$ 10–15%). The size of the effect will be limited in a given tumour by the intercapillary distances. In a tumour where these distances are large most viable tumour will be in the form of cords so that even under very well oxygenated conditions cord radius will be able to increase substantially without overlapping with adjacent structures. Where the capillaries are closer together, improved oxygenation with cause cord expansion and overlap so that they will not be inlcuded in our scoring method which relies on the presence of necrosis to delineate the limit of oxygen diffusion. In the CaRH a substantial part of the tumour volume is composed of corded stuctures within necrosis though there are areas, particularly at the periphery where necrosis is sparse or absent. Thus, it would be inaccurate to state that the mean oxygen diffusion distance in this tumour is the same as the mean cord radius (105 ± 2 µm); it will actually be smaller. All we can say is that this value is proportional to the diffusion distance within the corded region of the tumour. Another consequence of this will be a tendency to underestimate diffusion distance when tumour oxygenation is improved and to overestimate it when oxygenation is impaired.

Cord radius was reduced by 12% in animals exposed to 10% $O_2$ for 48 h. This coincides exactly with a 12% reduction in radius reported by Tannock (1970) in a mouse mammary carcinoma under the same conditions, though the control value was only 85 µm in that study. What does this tell us about the relationship between the arterial blood oxygen tension and the oxygen available at depth in the tumour? This will be dependent on several factors, but one of the most important is the binding affinity of haemoglobin for oxygen. In mouse blood a drop in inspired $pO_2$ from about 150 mmHg to 70 mmHg reduces the oxygen carried in the blood by about half, because the $Hb/O_2$ dissociation curve is steep over that range – the effect would be much less in man where the haemoglobin binding affinity is much higher. The next step is to consider how the radial distance for oxygen diffusion will vary with the $pO_2$ in the supplying vessel. An analysis of this relationship has recently been published (Groebe & Vaupel, 1988) which allows some estimation of how the range for $O_2$ diffusion would be expected to fall for a drop of 50% in the arterial $pO_2$. We find that the reduction in diffusion distance is very dependent on the absolute $pO_2$ in the supplying vessel so that a fall from 100 mmHg to 50 mmHg reduces the distance by 26% whereas a drop from 20 mmHg to 10 mmHg reduces it by 38%. The vascular network is, of course, a three dimensional structure so we need to consider $pO_2$ changes along the length of the supplying vessel. The distribution of cord radii in our tumours under all conditions was very wide (10–200 µm). This heterogeneity is probably dominated by two main effects. Firstly, the radius must fall along the length of the supplying vessel from the arterial to the venous end as $pO_2$ falls and secondly, the blood flow within individual vessels will differ. Both effects will contribute to the distributions observed though it seems likely that where cords are very narrow the blood flow within that vessel must be severely impaired. A detailed quantitative analysis of the data is not possible, however, because the two dimensional scoring method can yield some spuriously high values for cord radius where vessels are hidden above or below the plane of section. It is possible, however, to detect some interesting characteristics of the frequency distributions. It is evident (Figure 3) that the proportion of very narrow cords ($<$70 µm) is significantly lower in 100% $O_2$ compared with air, though there is no increase in this proportion in 10% $O_2$. The major change in the size distribution on reducing the inspired $pO_2$ to 10% is a reduction in the proportion of cords in the 100–170 µm range.

This suggests that it is areas of relatively good perfusion that are most vulnerable to $pO_2$ reduction while cords in all size ranges appear to benefit from an increase in oxygenation. There is also a suggestion (Figure 3, top panel) that it may be possible to reach a maximum radius (in this tumour about 200 μm) beyond which cords will not grow even if more $O_2$ is made available. This could represent the point at which the diffusion of other substrates such as glucose become limiting for cell survival.

The impact of raised or lowered arterial $pO_2$ on oxygen diffusion distances has also been modelled by Groebe and Vaupel (1988). The numbers they derive rely on the exact values chosen for oxygen consumption, $Hb/O_2$ binding affinity, intercapillary distance and capillary length. Nevertheless, the diffusion radii they obtain for the normal situation (mean of arterial and venous end of vessel = 62 μm) or at an arterial $pO_2$ of 50 mmHg which will be close to that found in mice breathing 10% $O_2$ (mean = 49 μm) are considerably lower than those for cord radius in our study. We should be aware, however, that the point at which $O_2$ is reduced to less than 1 mmHg (the cut off chosen by Groebe & Vaupel, 1988) and the point at which tumour cells die could be quite different even if oxygen is the critical nutrient. The discrepancy arises because cells will continue to survive in hypoxia for some time. Clamping experiments have shown that 50% of the cells in a mouse sarcoma are dead after 8 h of total nutrient deprivation (Denekamp *et al.*, 1983) and preliminary data for the CaRH indicate that this survival time lies between 6 and 16 h (Hill, personal communication). It seems reasonable to assume that cell survival time in an unclamped tumour will not be less than under this most extreme form of nutrient deprivation so, for the purpose of this discussion, we will assume that the CaRH cells remain morphologically intact for at least 8 h after they pass the $O_2$ diffusion limit, during which time they will have migrated, based on our previous labelling studies with the RH carcinoma (Hirst *et al.*, 1982), by about 16 μm. Thus we would always expect that cord radii will exceed the $O_2$ diffusion distance by a considerable margin, creating the population of 'chronically' hypoxic cells originally proposed by Thomlinson and Gray (1955).

Our data clearly demonstrate that tumour cord radius is an indicator of oxygen availability within the tumour and may have some prognostic value, particularly if other characteristics such as hypoxia tolerance and $O_2$ consumption rates can be established *in vitro* in cells from the same tumour. There have been several attempts to correlate morphological parameters of presumed significance to tissue oxygenation, with the outcome of radiotherapy of human tumours (Awwad *et al.*, 1986; Lauk *et al.*, 1989; Siracka *et al.*, 1982). The conclusions from these studies were apparently inconsistent, one (Lauk *et al.*, 1989) actually showing a highly significant direct correlation between the mean distance of tumour cells from the nearest blood vessel and local control by radiotherapy of oral squamous cell carcinomas. A large mean distance from tumour cells to blood vessel must mean either that the vessel was supplying a lot of oxygen (i.e. high blood flow), $O_2$ consumption by the tumour cells was low or that the tumour cells have a high tolerance from hypoxia. This last possibility would create a high hypoxic fraction though the first two would tend to decrease it. Also for purely geometric reasons the hypoxic rim of cells around a large cord is smaller as a proportion of the total volume than that surrounding a small one. There are clearly difficulties in using purely morphologic parameters as prognostic indicators, but if we focus only on relative differences within the same tumour before and after a particular intervention, information useful to therapy could be obtained.

We have chosen in this analysis to focus on oxygen as the substrate whose manipulation has important consequences for tumour cell survival and diffusion distances. This is probably an oversimplification of the complexity of tumour metabolism in view of the fact that we did not measure glucose levels in the tumour or even in the blood. It has been shown, however, in a tumour spheroid model *in vitro* that the thickness of the viable rim of tumour cells surrounding necrosis is only sensitive to glucose levels when they fall to less than half the normal blood level of 60–130 mg 100 $ml^{-1}$ (Tannock & Kopelyan, 1986). We conclude, therefore, that the changes in morphology reported in the present study can be attributed predominantly to the effects of oxygen, though experiments are in progress to determine the importance of glucose.

Our data support the view that cord shrinkage is a major component of the adaptation (Hirst, 1986) proposed to account for changes in radiosensitivity (Siemann *et al.*, 1979) after reduced or elevated oxygen availability. They do not, however, suport this as a mechanism for adaptation to reduce haematocrit as previously suggested (Hirst, 1986; Hirst & Wood, 1987). We do not yet have data showing this radiobiological adaptation in the RH carcinoma used in the present study, though it is perhaps surprising that low haematocrit (<30%) blood can support the same cord radius as normal blood. It suggests that the reduced viscosity, and improved tissue perfusion, compensates for the reduced oxygen carrying capacity (Sevick & Jain, 1989). We have no evidence for this in the RH carcinoma but our studies of the NT carcinoma show a reduction in relative perfusion after anaemia compared with the expected increase seen in normal tissues (Sensky & Hirst, unpublished).

Finally, our data (Figure 2) show clearly that improved oxygenation very quickly (<3 h) leads to a small but significant ($P<0.05$) increase in cord radius, presumably through cell growth or proliferation. This rapid response is perhaps surprising in view of the relatively long potential doubling time (58 h) measured in this carcinoma (Hirst *et al.*, 1982) and suggests that cell growth rather than simply proliferation may be involved in cord expansion. Whatever the mechanism, this observation emphasises that if methods are to be used to improve tumour oxygenation during radiotherapy they should be applied as briefly as possible.

This work is supported entirely by the Cancer Research Campaign. We wish to thank Peter Russell and his staff for production and care of the mice used in these experiments.

### References

AWWAD, H.K., EL NAGGAR, M., MOCKTAR, N. & BARSOUM, M. (1986). Intercapillary distance measurement as an indicator of hypoxia in carcinoma of the cervix uteri. *Int. J. Radiat. Oncol. Biol. Phys.*, **12**, 1329.

DENEKAMP, J., HILL, S.A. & HOBSON, B. (1983). Vascular occlusion and tumour cell death. *Eur. J. Cancer Clin. Oncol.*, **19**, 271.

GROEBE, K. & VAUPEL, P. (1988). Evaluation of oxygen diffusion distances in human breast cancer xenografts using tumor-specific *in vivo* data: role of various mechanisms in the development of tumor hypoxia. *Int. J. Radiat. Oncol. Biol. Phys.*, **15**, 691.

HIRST, D.G. (1986). Anemia: a problem or an opportunity in radiotherapy? *Int. J. Radiat. Oncol. Biol. Phys.*, **12**, 2009.

HIRST, D.G., HAZELHURST, J.L. & BROWN, J.M. (1984). The effect of alterations in haematocrit on tumour sensitivity to X-rays. *Int. J. Radiat. Biol.*, **46**, 345.

HIRST, D.G. & WOOD, P.J. (1987). The adaptive response of mouse tumours to anaemia and retransfusion. *Int. J. Radiat. Biol.*, **51**, 597.

HIRST, D.G., DENEKAMP, J. & HOBSON, B. (1982). Proliferation kinetics of endothelial cells in three mammary carcinomas. *Cell Tissue Kinet.*, **15**, 251.

HLATKY, L., SACHS, R.K. & ALPEN, E.L. (1988). Joint oxygen-glucose deprivation as the cause of necrosis in a tumor analog. *J. Cell. Physiol.*, **134**, 167.

LAUK, S., SKATES, S., GOODMAN, M. & SUIT, H.D. (1989). A morphometric study of the vascularity of oral squamous cell carcinomas and its relation to outcome of radiotherapy. *Eur. J. Cancer Clin. Oncol.*, **25**, 1431.

MUELLER-KLIESER, W., FREYER, J.P. & SUTHERLAND, R.M. (1983). Evidence for a major role of glucose in controlling development of necrosis in EMT6/Ro multicell tumor spheroids. In *Oxygen Transport to Tissue*, Bicher, H. & Bruley, D.F. (eds), p. 487. Plenum Publishing Corp: New York.

SEVICK, E.M. & JAIN, R.K. (1989). Viscous resistance to blood flow in solid tumours: effect of hematocrit on intratumor blood viscosity. *Cancer Res.*, **49**, 3513.

SIEMANN, D.W., HILL, R.P., BUSH, R.S. & CHHABRA, P. (1979). The *in vivo* radiation response of an experimental tumour: the effect of exposing tumour-bearing mice to a reduced oxygen environment prior to but not during irradiation. *Int. J. Radiat. Oncol. Biol. Phys.*, **5**, 61.

SIRACKA, E., SIRACKY, J., POPPOVA, N. & REVESZ, L. (1982). Vascularization and radiocurability in cancer of the uterine cervix. A retrospective study. *Neoplasma*, **29**, 183.

TANNOCK, I.F. (1970). Effects of $pO_2$ on cell proliferation kinetics. In *Time and Dose Relationships in Radiation Biology as Applied to Therapy*, Bond, V.P., Suit, H.D. & Marcial, V. (eds), p. 215. Brookhaven, National Laboratory: Upton.

TANNOCK, I.F. & KOPELYAN, I. (1986). Influence of glucose concentration on growth and formation of necrosis in spheroids dervived from a human bladder cancer cell line. *Cancer Res.*, **46**, 3105.

THOMLINSON, R.H. & GRAY, L.H. (1955). The histological structure of some human lung cancers and the possible implications for radiotherapy. *Br. J. Cancer*, **9**, 539.

TROTT, K.-R. (1983). Die Bedeutung de Besonderheiten der Tumormikrozirklation fuer die Strahlentherapie. *Mikrozik. Forsch. Klin.*, **2**, 114.